# Defining Competencies for Policymaking in Public Health: A Scoping Review on the State-of- The-Art

**DOI:** 10.3389/phrs.2026.1609031

**Published:** 2026-05-14

**Authors:** Lorraine Frisina Doetter, Gabriela de Carvalho, Emilia Aragon de Leon

**Affiliations:** 1 Faculty of Public Health, Universitat Bremen, Bremen, Germany; 2 Institute of Government and Public Policy (IGOP), Universitat Autonoma de Barcelona, Barcelona, Spain; 3 Policy and Governance for Health, World Health Organization Regional Office for Europe, Copenhagen, Denmark

**Keywords:** competencies, framework, policymaking, public healh, state-of-the-art

## Abstract

**Objectives:**

This study examines the competencies required for effective public health policymaking, seeking to identify and catalog existing scholarship and to develop an analytical framework organizing competencies into cross-cutting domains to guide training and capacity development.

**Methods:**

A scoping review of the literature was conducted, analysing 43 peer-reviewed studies that addressed policymaking competencies in public health. Competencies were inductively coded, synthesized, and organized into themes using thematic analysis.

**Results:**

64 competencies were identified and mapped into eight domains: analytical and research, political, leadership and management, design thinking, collaboration and networking, communication, resource mobilization, and technology readiness. Analytical and research competencies were most frequently cited, underscoring the centrality of evidence-based decision-making. Design thinking and collaboration also featured prominently, reflecting the growing emphasis on complexity management and stakeholder engagement. By contrast, communication, resource mobilization, and technology readiness were underrepresented, despite their importance for modern policymaking.

**Conclusion:**

The resulting eight-domain framework consolidates a fragmented field and underscores the need for more comprehensive competency-building strategies. It offers practical guidance for policymakers, educators, and institutions seeking to strengthen public health leadership.

## Introduction

Public health policy-making can be understood as the multi-actor, iterative process through which governments and institutions formulate, implement, and evaluate decisions, laws, and actions aimed at improving population health, drawing on evidence, political considerations, and societal values [1, 2]. It is distinct from healthcare policy, which focuses primarily on the organisation, financing, and delivery of clinical services, and from the broader field of public health, which encompasses surveillance, research, and professional practice beyond the policy process itself [3, 4]. Policymakers operating within this complex field must regularly draw on their knowledge and skills–or their *competencies–*for effective policymaking. This includes abilities to lead and facilitate engagement in and between policy sectors and with the public, to navigate the larger landscape of public health institutions and governance levels, to generate, understand, and apply evidence for decision making, as well as to negotiate the demands of diverse stakeholders. These policymaking demands give rise to fundamental questions: what are the competencies needed to fulfill such multifaceted roles? What thematic domains of competencies emerge from the literature on public health policymaking?

Answering these questions is critical in light of the growing strain on public health systems, driven by demographic aging, the burden of non-communicable diseases, and shocks such as pandemics, environmental crises, and geopolitical instability. Evidence across countries indicates that the quality of public health policy is closely linked to the effectiveness of public health leadership—demonstrated not only during the COVID-19 pandemic [5, 6] but also in non-emergency contexts [7]. These developments underscore the urgency of investing in training that equips policymakers to make informed decisions, identify problems accurately, and design and implement data-driven, contextually appropriate solutions.

This study examines current scholarship on the competencies essential for effective public health policymaking. Competencies are generally understood as observable abilities reflecting the integration of knowledge, skills, and attitudes directed toward the performance of tasks [8, 9]. They are often transversal in nature, applicable across activities and adaptable to diverse contexts [10–12]. Two main approaches dominate the literature: the behavioral approach, which defines competencies as underlying characteristics—knowledge, motives, traits, self-image, social roles, and skills—linked to effective performance; and the functional approach, which emphasizes observable task performance against occupational standards [12]. In practice, behavioral competencies reflect intrinsic qualities that, when applied to tasks, manifest as measurable functional competencies. Defining competencies in this way matters because they are widely regarded as malleable. With appropriate training and support, they can be developed and strengthened, yielding measurable improvements in performance; conversely, without continued investment, they risk erosion over time [13–15].

Against this backdrop, the present study undertakes a scoping review and synthesis of the literature to clarify the competencies required for effective public health policymaking. Its objectives are twofold: first, to identify and catalog existing literature on competencies for public health policymaking; and second, to develop an analytical framework organizing competencies into cross-cutting domains to inform future research, policy design, and training.

## Methods

### Study Design

**FIGURE 1:**
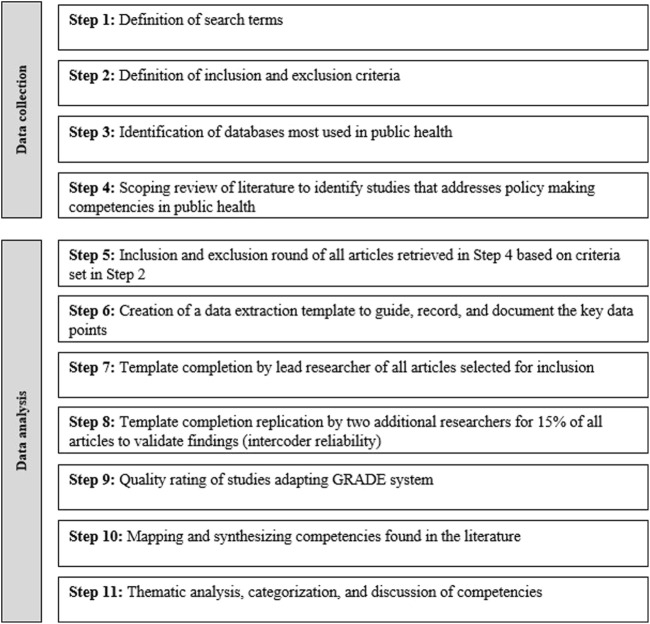
Flowchart of methodological process (Scoping review, global, 2024). (Source: Own presentation).

To analyze extant scholarship on competencies for public health policymakers, we conducted a scoping review of the literature, a method best served to map knowledge, identify gaps, and clarify concepts [16, 17]. The study, first, takes inventory of the literature that addresses competencies of relevance to policymaking in public health. The retrieved scholarship is then examined to synthesize identified skills to understand their significance in public health policymaking. Following the approach put forth by Altheide [18] and Bowen [19] and further developed by Sternkopf et al. [20], this assessment follows an eleven-steps methodological process represented in Figure 1.

### Data Sources and Search Strategy

The scoping review aimed to identify existing literature on competencies related to policymaking in public health. The search was conducted using a clearly defined set of terms, as well as inclusion and exclusion criteria to ensure reproducibility and transparency. To synthesize as much evidence as possible, we did not limit our search by any specific observation period, including all relevant literature regardless of publication date or time frame, nor by any specific geographical region. Searches were performed in the English language. The searches were conducted between February and March 2024 in the well-known and indexed public health databases Cochrane Library, PubMed, Science Direct, and Web of Science. To capture non-indexed but relevant studies, Google Scholar was also consulted. Search results were imported and deduplicated using Zotero reference management software. Notably, only peer review publications were included in the analysis, as well as studies that explicitly addressed policy making competencies. This led to the exclusion of frameworks aimed at public health practitioners or professionals.

### Search Terms

Search terms were defined based on the research goal and limited, at first, to title, keywords, and abstract: “policymaking” OR “policy making” OR “policy develop*” OR “policy design” OR “policy formulat*” OR “policy implementat*” OR “policy evaluat*” OR “policy decision mak*” OR “policy agenda setting” AND “competencies” OR “skills” OR “capabilit*” AND “public health” OR “health policy”. Search terms were adjusted to each database. These are displayed in Supplementary Appendix 1.

### Eligibility Criteria

We analyzed all retrieved results, except on Google Scholar, where we reviewed the articles contained in the 10 first pages given the lower precision and lack of advanced filtering in the database. After removing duplicates and papers without abstracts, the searches resulted in 416 hits in total. Title and abstracts were then carefully screened for inclusion on the basis that articles (a) focus specifically on (public) health policies or public policies in general, (b) address specifically policymakers, either alone or in combination with other actors (e.g., health professionals), and (c) focus, whether explicitly or implicitly, on any stage of policymaking (agenda-setting, formulation, adoption, implementation, evaluation, and termination). Doing so, we exclude research that focuses on public policies unrelated to health (e.g., energy, housing, etc.), that does not deal with policymaking, that only addresses healthcare practitioners without referring to policymakers, that focuses only on tools for acquiring competencies (e.g., software and platforms) or addresses competencies gained during education (e.g., curriculum analysis), and that cover topics outside the defined scope, such as clinical trials. All the listed inclusion criteria are necessary conditions, but not sufficient on their own. On the other hand, any of the exclusion criteria are sufficient to exclusion.

### Data Characterization

**FIGURE 2:**
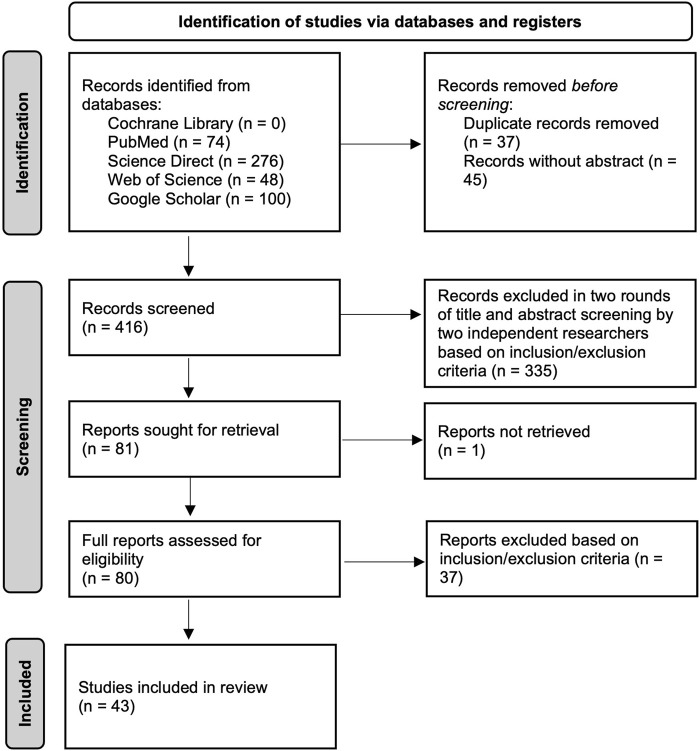
PRISMA flow diagram (Scoping review, global, 2024). (Source: Page et al. [21]).

A PRISMA flow diagram of the review process (Figure 2) is presented. For a full included listing of articles, see Supplementary Appendix 2.

To support the analysis of the retrieved literature, a structured data extraction template was developed using a deductive approach (see Annex 3). This template captured key dimensions including metadata, country focus, policymaking level (e.g., national, subnational), policymaking stage(s) addressed (e.g., agenda setting, formulation, implementation, evaluation), and the specific competencies referenced.

### Data Summary and Synthesis

All articles selected for inclusion were analyzed thoroughly by a lead researcher who also extracted data into the template to guide the systematic identification and recording of key data points (completed templates are available upon request). Two additional researchers replicated this process for 15% of all articles to validate the findings and ensure coder reliability. The articles selected for verification were randomized.

### Data Analysis

Through our scoping review, we identified a total of 64 unique competencies from the retrieved literature. We followed Braun and Clarke [22] guidelines for thematic analysis, involving familiarization with the data, initial coding, searching for themes among coded segments, reviewing themes for coherence and distinctiveness, defining and naming themes clearly, and finally categorizing competencies into thematic domains.

We mapped and synthesized evidence found in the scoping review using thematic analysis. This qualitative analytical approach allows researchers to identify, analyze, and report recurring themes and patterns in the data in a systematic and transparent way [22, 23]. Our thematic analysis followed an inductive approach. The final set of themes was defined, named, and organized systematically, providing a structured synthesis of the competencies identified in the literature. Following this, the quality of the studies was evaluated following the GRADE scheme in line with Pieper et al. [24].

Based on our thematic analysis, we developed an analytical framework to systematically organize the identified competencies required for effective public health policymaking. The framework classifies competencies into inductively-derived thematic domains, each capturing specific areas of knowledge and measurable skills.

## Results

**FIGURE 3:**
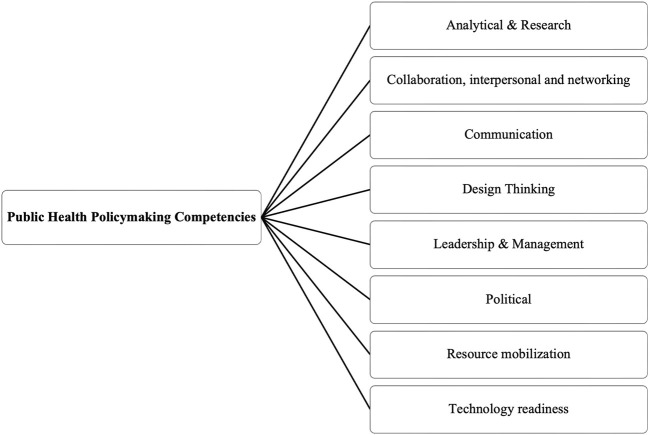
Analytical framework for mapping competencies in public health policymaking (Scoping review, global, 2024). (Source: Own presentation).

Following a process of screening conducted by three senior researchers, 43 articles were selected for final inclusion. The analytical framework developed in this study (Figure 3) systematically organizes the identified competencies into eight thematic domains. In what follows, the results of this mapping exercise are presented, alongside illustrative examples from the included studies.

### Competencies for Effective Public Health Policymaking

We define competencies as observable abilities integrating knowledge, skills, and attitudes directed at performing specific tasks that are applicable across multiple activities, which can be developed or eroded over time depending on contextual conditions such as training availability and practice opportunities. These competencies were inductively categorized into eight clearly defined dimensions, each reflecting particular knowledge areas and skills critical to public health policymaking: (a) analytical and research, (b) political, (c) leadership and management, (d) design thinking, (e) collaboration, interpersonal, and networking, (f) communication, (g) resource mobilization, and (h) technology readiness. The domains, the specific competencies by domain, as well as the studies that these appear are presented in Table 1.

**TABLE 1:** Identified competency domains and competencies by study (Scoping review, global, 2024).

Domain	Competency	Articles
Analytical & research	Ability to apply analytical and research skills to generate and utilize evidence for policymaking	[25–43]
	Ability to develop, adapt, and utilize analytical tools and logic models tailored to specific policy contexts	[25, 35, 44–46]
	Ability to integrate contextual information into policy analysis	[25, 26, 31, 35, 41, 46–50]
	Ability to recognize and balance different methodological strategies	[47, 51]
	Ability to apply knowledge about competency frameworks	[26, 52]
	Ability to translate knowledge about equity to policymaking	[53]
	Ability to engage in knowledge translation for policy formulation	[39, 54, 55]
Design thinking	Ability to engage in policy design thinking	[31, 44, 46, 56]
	Ability to utilize imaginative approaches to policymaking	[29, 46, 57]
	Ability to design and utilize feedback mechanisms across the policy cycle	[46, 58]
	Ability to engage in foresight for policy formulation	[29, 30, 51]
	Ability to make use of past learnings for experiential policymaking	[29, 41, 49, 59]
	Ability to accommodate complexity in policymaking	[31, 44, 45, 49, 56, 57]
	Ability to engage in operational thinking	[35, 44–46, 60]
	Ability to apply analytical skills and operational thinking to perform policy functions	[46]
	Ability to diagnose policy problems, identify root causes, and generate context-appropriate solutions	[31, 35, 43, 44, 46, 49, 50]
	Ability to define policy problems clearly	[41, 43, 50]
	Ability to evaluate and continuously improve policy interventions	[27, 28, 42, 43, 49, 58, 61]
Collaboration, interpersonal & networking	Ability to build and leverage networks and partnerships to guide evidence-informed policymaking	[25, 28, 29, 40, 56, 62, 63]
	Ability to build trust	[40, 64]
	Ability to establish and collaborate with interdisciplinary teams	[41, 44]
	Ability to engage and collaborate with stakeholders	[25, 40, 41, 43, 45, 51, 56, 65]
	Ability to build and maintain a network	[51, 65]
	Ability to manage stakeholders	[65]
	Ability to identify and engage key actors	[44, 50, 51]
	Ability to mobilize and align different viewpoints and knowledge	[44, 47, 51, 65]
	Ability to collaborate with external partners	[43, 65]
	Ability to apply interpersonal skills for collaboration within and across organizations	[43, 44, 57, 65]
Political	Ability to cultivate and leverage political capital, authority, and influence	[43, 56, 65]
	Ability to build trust among stakeholders in the political system	[40, 56, 65]
	Ability to assess and enhance political acceptability and support	[40, 41, 43, 54, 56, 65, 66]
	Ability to apply political knowledge effectively throughout the policymaking process	[43, 65]
	Ability to identify policy champions	[39, 56, 67]
	Ability to create alliances with other political actors in the system	[44, 56, 59, 65]
	Ability to apply policy acumen involving knowledge about policy actors, positions, interests, resources, strategies	[33, 43, 46, 56, 65]
	Ability to apply knowledge of relevant legislation and regulations in policy actions	[46, 53, 65]
Leadership and management	Ability to lead during a public health emergency	[61, 65]
	Ability to establish and manage teams	[48, 60, 62, 65]
	Ability to manage conflicts	[57, 65]
	Ability to lead with strategic vision and manage resistance	[40, 48, 63, 65]
	Ability to manage human, financial, and infrastructural resources	[43, 44, 65]
	Ability to lead for a positive research climate in an organization	[35]
	Ability to lead change in public behavior and attitudes	[62]
	Ability to lead with accountability	[39]
	Ability to prioritize competencies for policymaking staff	[25, 26, 39, 61]
Communication	Ability to develop concise and effective policy briefs	[25, 41]
	Ability to persuade through framing and communication of evidence	[43, 59, 64]
	Ability to engage in purposeful communication	[43, 56, 64]
	Ability to use multiple policy languages for persuasion	[43, 56, 64]
	Ability to understand and utilize targeted communications and evidence summaries	[25, 56]
	Ability to apply communication, negotiation, and consensus-building skills	[40, 43, 56, 64]
Resource mobilization	Ability to mobilize resources for policy development and implementation	[33, 43, 44, 65]
	Ability to manage and allocate financial resources	[33, 37, 43, 44, 65]
	Ability to identify and leverage funding opportunities	[33, 65]
	Ability to identify and manage resources adequately	[43, 44, 65]
Technology readiness	Ability to understand and make use of AI tools for policy design	[68, 69]
	Ability to understand and implement cybersecurity measures	[69]
	Ability to understand and use big data	[37, 68, 69]
	Ability to utilize new data technologies and big data analytics	[37, 51, 68, 69]
	Ability to leverage science, technology, and innovation in society	[30, 51]

Source: Own compilation.

In terms of frequency across the reviewed literature, *analytical and research* competencies emerge as the most prominent domain, referenced in 72% of the included articles. This strong emphasis suggests a continued prioritization of skills such as data interpretation, evidence appraisal, and policy-relevant analysis—all core capabilities for evidence-informed public health policymaking. The next most frequently cited domain is *design thinking* (48%), reflecting a growing interest in problem-framing, creative solution-building, and systems-oriented approaches that accommodate the complexity of policymaking processes. *Collaboration, interpersonal, and networking* competencies are mentioned in 37% of the papers, underlining the recognized importance of trust-building, multi-stakeholder engagement, and effective coordination across institutional boundaries. *Political* competencies, found in 32.5% of studies, highlight the need to understand power dynamics, stakeholder interests, and the policy environment. *Leadership and management* competencies were also identified in 28% of studies, particularly in relation to strategic direction, team management, and implementation oversight.

By contrast, competencies related to *communication* (16.3%), *resource mobilization* (11.6%) and *technology readiness* (11.6%) were referenced less, however, with clear recognition, perhaps more recently, of their critical importance in effective policy-making.

A temporal reading of the included studies—which span from 2008 to 2023, with a mean publication year of 2017.5 — reveals that not all domains have been equally prominent across time. *Analytical and research* competencies are the most established in the literature, with references dating back to 2008 and appearing consistently throughout the entire review period. *Design thinking* and *collaboration, interpersonal, and networking* competencies began emerging in the early 2010s but grew substantially after 2015, reflecting a gradual shift in the literature toward more systemic and relational approaches to policymaking. *Political* competencies, despite their practical centrality, appear almost exclusively in post-2015 studies, with only one reference pre-dating that threshold. *Resource mobilization* is the most strikingly recent domain—entirely absent from the literature before 2016 — suggesting it has only recently been recognized as a distinct competency area. *Technology readiness* follows a similar pattern: while one early study from 2011 touched on science and innovation foresight, the competencies most specific to this domain—AI tools, big data analytics, and cybersecurity—appear exclusively in 2023 publications, reflecting the very recent emergence of digital transformation as a policy concern. *Leadership and management*, and *communication* competencies are present throughout the review period but with a clear concentration of studies in the post-2015 window. *Analytical and research competencies* involve the application of analytical methods and conducting research tailored to public health policymaking. These competencies enable policymakers to systematically utilize evidence, engage effectively with research methodologies, and apply analytical insights to complex policy issues. This domain comprises seven competencies out of the 64 identified across all reviewed studies and is the most referenced domain. The most frequently cited competency in this domain is the *ability to apply analytical and research skills to generate and utilize evidence for policymaking*, referenced in 19 different studies. For instance, one study notes that it is imperative to “develop policy in an evidence-based fashion, i.e., using knowledge produced by a diverse group of professions and the best empirical information available” ([61] (p588)). The second most frequently cited competency is the *ability to integrate contextual information into policy analysis*, as one article explains: “evidence should be used in conjunction with contextual factors, including public opinion, equity, feasibility of implementation, affordability, sustainability, and acceptability to stakeholders” ([26] (p4)). Other competencies in this domain include the *ability to recognize and balance different methodological strategies and apply and translate knowledge*. These competencies collectively underpin policymakers’ ability to leverage evidence effectively and navigate complex informational environments. Their frequent appearance in the literature underscores the centrality of research capacity for effective policymaking.

While analytically-oriented competencies focus on working with evidence—its generation, appraisal, and translation into policy-relevant knowledge—*design thinking* competencies are concerned with working with problems: diagnosing root causes, imagining context-appropriate solutions, accommodating complexity, learning iteratively from experience, and engaging in foresight. A policymaker may be highly proficient in evidence appraisal and yet lack the capacity to creatively frame a problem or adapt a solution to a changing context. These are distinct and complementary repertoires, and their separation into two domains reflects this difference in orientation. In contrast, *design thinking* competencies refer to the capacity to apply innovative, creative, and strategic thinking to address challenges in public health policymaking. These competencies help policymakers creatively solve problems, adapt to complexities, and iteratively improve policy interventions. This domain includes 11 competencies. Among the most referenced ones is the *ability to diagnose policy problems, identify root causes, and generate context-appropriate solutions*, with one article highlighting that the foundation of policymaking is the “proper definition of the policy problem”, stressing the importance of having a clear and unambiguous understanding before advancing to subsequent stages of policy development ([41] (p5)). The *ability to accommodate complexity in policymaking* is also widely cited, reflecting the need to navigate dynamic systems and uncertainty, as well as *the ability to evaluate and continuously improve policy interventions.* Less frequently but notably, studies refer to *foresight*, *experiential learning*, and *feedback mechanisms* across the policy cycle. Collectively, these competencies equip policymakers to iterate and adjust policy solutions in response to challenges.

*Collaboration, interpersonal, and networking* competencies encompass skills required to effectively engage, build relationships, and collaborate and mobilize within and beyond organizational boundaries in policymaking contexts. These competencies are pivotal in mobilizing collective efforts, leveraging diverse expertise, and aligning various stakeholders towards common public health goals. This domain includes ten competencies and appears in 15 studies. The most frequently cited competencies are the *ability to engage and collaborate with stakeholders*—stressed in one study as crucial to “address key policy questions” ([25] (p2))—and the *ability to build and leverage networks for evidence-informed policymaking*. Other commonly mentioned skills include *collaborating with interdisciplinary teams, identifying and engaging key actors*, and *applying interpersonal skills for collaboration across organizations*. While competencies such as *stakeholder management* and *network maintenance* are less frequently noted, they underscore the operational depth required for sustained partnership building. Taken together, these point to the vital role of relational capacity in enabling implementation of collective policy efforts and knowledge exchange.

*Political competencies* comprise the capacity to navigate and effectively influence political contexts and dynamics relevant to public health policymaking. These competencies allow policymakers to build necessary relationships, leverage political resources, and effectively engage within complex political environments. This domain comprises eight competencies, referenced across 14 studies. The most frequently cited is the *ability to assess and enhance political acceptability and support*, with a study saying that this is “one of the first critical issues that must be taken into cognizance in policy development” ([41] (p7)). *The ability to apply policy acumen involving knowledge about policy actors, positions, interests, resources, strategies* is also relevant: “policy acumen, consisting of insights about positions, interests, resources and strategies of key players in the policy process, and the practical implications of policy actions, forms the basis for actors to make sound judgment on the desirability and feasibility of different policies” ([43] (p8)). Additional competencies point to the *capacity to apply political knowledge effectively*, *identify policy champions*, and *build trust within political systems*. These skills position policymakers to anticipate and respond to the shifting dynamics of political contexts, aligning policy objectives with political opportunity structures to enhance prospects.

*Leadership and management competencies* entail the abilities required to guide teams, manage resources effectively, and lead organizations strategically within public health policymaking. Competencies in this domain highlight their fundamental role in coordinating efforts and ensuring effective policy implementation. This domain includes nine distinct competencies, most prominently the *ability to establish and manage teams* and to *prioritize competencies for policymaking staff* referenced in four studies. Examples from the literature also include the *ability to lead during public health emergencies* and *manage conflicts*. Further emphasized were competencies like leading with strategic vision and managing resistance, and managing human, financial, and infrastructural resources effectively. These abilities are critical in ensuring continuity, motivation, and direction within policymaking institutions.

*Communication competencies* comprise the essential skills for clearly conveying information, persuading diverse audiences, and effectively facilitating dialogue and consensus in public health policymaking contexts. The six competencies identified in this domain were frequently identified as crucial for bridging knowledge gaps, aligning stakeholder perspectives, and ensuring successful policy advocacy and implementation. Notably, literature highlighted competencies such as the *ability to craft concise and effective policy briefs, persuade through effective framing of evidence,* and *utilize targeted communications and evidence summaries* to facilitate informed policymaking.

*Resource mobilization*, while less frequently cited than other domains, represent essential pillars of effective public health policymaking. The domain includes four competencies, most commonly the *ability to manage and allocate financial resources* and *to mobilize support for policy implementation*—each referenced in four studies. These skills ensure that policymakers can identify, secure, and deploy financial and material assets to operationalize policy goals. *Technology readiness*, comprising five competencies, is also among the least referenced domains. Nonetheless, studies underscored its rising relevance, particularly the *ability to utilize new data technologies*, such as AI and big data analytics, and *to implement cybersecurity measures to protect policy infrastructure*. These competencies are critical in an increasingly digital policy environment. Their limited presence likely reflects the novelty and ongoing evolution of technological applications in public health in general [70], as well as in policymaking.

## Discussion

This study mapped 64 competencies relevant to public health policymaking, synthesizing them into eight thematic domains: analytical and research, political, leadership and management, design thinking, collaboration and networking, communication, resource mobilization, and technology readiness. Together, these domains highlight the breadth of skills and knowledge needed to navigate the increasingly complex environment of public health policy. The analysis demonstrates that while policymakers are often expected to combine technical, political, and interpersonal abilities, the emphasis in the literature has been uneven, with certain domains dominating the discourse while others remain underexplored.

The prominence of analytical and research competencies, cited in nearly three-quarters of reviewed studies, reflects the continuing centrality of evidence-based policymaking in research. The ability to appraise and apply data, balance methodological strategies, and integrate contextual factors has long been recognized as foundational to public health leadership, and our findings reaffirm this priority. At the same time, the growing attention to design thinking and collaboration signals an important shift. Policymakers are increasingly expected to diagnose complex problems, accommodate uncertainty, and co-create solutions through multi-stakeholder engagement—competencies that move beyond evidence appraisal to problem-framing, systems thinking, and relational capacities. However, the dominance of analytical and research competencies warrants critical reflection, and may point to a mismatch between the literature and the demands of practice. Researchers may naturally gravitate toward competencies closer to their own professional world—evidence generation and appraisal—while the political, communicative, and relational dimensions that are equally, if not more, demanding in practice remain understudied. The growing complexity of public health policy—marked by rising political contestation, resource constraints, and digital transformation—suggests that the lesser cited competencies may be more critical in practice than their representation in the literature implies. The temporal distribution of the included studies adds a further layer to this picture. The 43 reviewed studies span 2008 to 2023, and a reading of when different domains emerged in the literature may suggest that scholarly attention to competencies has evolved in response to broader societal and institutional shifts. Analytical and research competencies have been consistently studied throughout the entire period, confirming their longstanding centrality. However, domains such as resource mobilization and technology readiness are almost exclusively post-2015 phenomena—resource mobilization is entirely absent before 2016, while the most technology-specific competencies, relating to AI and big data, appear only in 2023. This lag between the emergence of real-world demands and their recognition in the scholarly literature raises a concern: if the competency framework reflects the literature as it stands today, it may already be underweighting capabilities that will become critical in the near future. This points to the need for periodic revision of competency frameworks to keep pace with the evolving demands of public health policymaking.

The practical importance of these underrepresented domains nonetheless warrants closer attention. Communication skills are central to framing evidence, shaping narratives, and aligning diverse interests—capacities that are essential for building the political support needed to advance public health agendas. Resource mobilization, while previously overlooked in mainstream competency frameworks, has become increasingly relevant given evolving financial pressures and governments deprioritizing financing for health [71]. Policymakers will need to rely more on their skills to effectively influence resource allocation for public health, as well as to identify alternative funding sources. Technology readiness, meanwhile, has been slow to appear in the literature, likely because scholarship in this area has historically focused on basic health sciences or clinical applications. Nevertheless, the rapid advances in Artificial Intelligence present both challenges and opportunities for public health policymaking—from summarizing large volumes of regulatory and scientific information to supporting evidence synthesis. Policymakers need to understand the appropriate use of these tools, while prioritizing equity, ethics, and risk management [70] A further contribution of this review is the recognition that many competencies are rooted in behavioral traits—such as political acumen, collaboration, and leadership—that translate into functional capabilities when applied in practice. This underscores the transversal nature of competencies: they cut across the policy cycle and are not fixed attributes, but rather developable abilities that can be cultivated or eroded depending on institutional environments, training opportunities, and organizational culture. This malleability reinforces the case for systematic investment in competency development as a means to strengthen both individual policymakers and the institutional capacity of public health systems.

At the same time, the study highlights notable gaps in the current scholarship. The limited attention to technology readiness and resource mobilization points to a lag between emerging demands on policymakers and the frameworks used to study and support them. Similarly, the fragmented way competencies are presented in the literature suggests the absence of a unifying model to guide both research and training specific to policy-making in public health. To address these gaps, the analytical framework proposed here provides a structured foundation for organizing competencies across domains and can serve as a practical tool for educators, policymakers, and institutions designing training and professional development programs.

This review is not without limitations. First, it reflects the scope and focus of the included studies, which may overemphasize certain domains while neglecting others. Moreover, the current scope does not account for research published in non-peer review journals or which focuses on competencies relevant for practitioners. Such literature may still speak to policymaker competency needs and are suggested as areas for further research to elaborate the present framework (see, e.g., [2, 72]). Second, the thematic synthesis, while systematic, is interpretive and subject to the biases inherent in qualitative analysis. Third, the dynamic nature of policymaking means that competency needs evolve with technological, political, and societal shifts, and future research will need to revisit and update these domains regularly. Fourth, while the methodology is sound, it has not been validated through stakeholder engagement to ensure it is contextually relevant. Future research should therefore prioritize stakeholder consultations—involving both educators and policymakers—to validate the 8-domain, 64-competency framework and ensure its contextual relevance across different health system settings. Stakeholders can also help to shed light on the competencies needed at specific stages of the policy cycle–an area largely neglected in current research.

In conclusion, this study provides the most comprehensive synthesis to date of competencies for effective public health policymaking. It identifies a rich but fragmented landscape, dominated by analytical and research skills but increasingly shaped by design thinking and collaboration. At the same time, it highlights the need to reinforce communication, resource mobilization, and technology readiness to prepare policymakers for contemporary and future challenges. By consolidating 64 competencies into eight domains, the study offers an analytical framework that can inform competency-building strategies at individual and organizational levels. For policymakers, educators, and institutions, these findings underscore the need to move beyond narrow technical training toward a holistic model of capacity development that equips public health leaders throughout their professional lives to navigate complexity, mobilize resources, and harness digital tools—ensuring that public health systems remain resilient, adaptive, and effective in the face of evolving global challenges.
