# Supplementary material for: Defining Competencies for Policymaking in Public Health: A Scoping Review on the State-of- The-Art
Source: Public Health Rev. 2026 May 14;47:1609031. doi: 10.3389/phrs.2026.1609031 (PMC13217404; doi:10.3389/phrs.2026.1609031)
Supplement: Supplementary file 1 [file Table1.docx]

**Appendix 1. Search commands by database**

| **Databases** | **Search terms** |
| --- | --- |
| Cochrane Library | ("policymaking" OR "policy making" OR "policy development" OR "policy design" OR "policy formulation" OR "policy implementation" OR "policy evaluation" OR "policy decision making" OR "policy decision making" OR "policy agenda setting") AND ("competencies" OR "skill" OR "capabilities") AND ("public health" OR "health policy") in Title Abstract Keyword |
| PubMed | ("Policymaking"[Title/Abstract] OR "Policy making"[Title/Abstract] OR "policy develop*"[Title/Abstract] OR "Policy Design"[Title/Abstract] OR "policy formulat*"[Title/Abstract] OR "policy implement*"[Title/Abstract] OR "policy evaluat*"[Title/Abstract] OR "Policy decision making"[Title/Abstract] OR "policy decision making*"[Title/Abstract] OR "Policy agenda setting"[Title/Abstract]) AND ("competenc*"[Title/Abstract] OR "Skill"[All Fields] OR "capabilit*"[Title/Abstract]) AND ("Public Health"[All Fields] OR "Health Policy"[Title/Abstract]) [All Fields] |
| Science Direct | Title, abstract, keywords: ("policy making" OR "policy developmen" OR "policy design") AND ("competencies" OR "skill" OR "capabilities") AND ("public health" OR "health policy") |
| Web of Science | ("Policymaking" OR "Policy making" OR "policy develop*"OR "Policy Design" OR "policy formulat*" OR "policy implement*"OR "policy evaluat*" OR "Policy decision making" OR "policy decision making*" OR "Policy agenda setting") AND ("competenc*" OR "Skill" OR "capabilit*") AND ("Public Health" OR "Health Policy") (Abstract) |
| Google Scholar | ("Policymaking" OR "Policy making" OR "policy develop*"OR "Policy Design" OR "policy formulat*" OR "policy implement*"OR "policy evaluat*" OR "Policy decision making" OR "policy decision making*" OR "Policy agenda setting") AND ("competenc*" OR "Skill" OR "capabilit*") AND ("Public Health" OR "Health Policy") |

Source: Own presentation.
