# Supplementary material for: Defining Competencies for Policymaking in Public Health: A Scoping Review on the State-of- The-Art
Source: Public Health Rev. 2026 May 14;47:1609031. doi: 10.3389/phrs.2026.1609031 (PMC13217404; doi:10.3389/phrs.2026.1609031)
Supplement: Supplementary file 2 [file Table2.docx]

**Appendix 2. Included Articles Scoping Review**

Ansell C, Geyer R. ‘Pragmatic complexity’ a new foundation for moving beyond ‘evidence-based policy making’? Policy Studies. 2016 Aug 16;1–19.

Armstrong R, Waters E, Dobbins M, Anderson L, Moore L, Petticrew M, et al. Knowledge translation strategies to improve the use of evidence in public health decision making in local government: intervention design and implementation plan. Implementation Sci. 2013 Dec;8(1):121.

Arroyo KK. Creative Policymaking: Taking the Lessons of Creative Placemaking to Scale. Artivate. 2017;6(2):58–72.

Barreto JOM, Romão DMM, Setti C, Machado MLT, Riera R, Gomes R, et al. An evidence-informed policymaking (EIPM) competency profile for the Brazilian Health System developed through consensus: process and outcomes. Health Res Policy Sys. 2023 Oct 12;21(1):105.

Bartlett W. Obstacles to Evidence‐based Policy‐making in the EU Enlargement Countries: The Case of Skills Policies. Soc Policy Adm. 2013 Aug;47(4):451–67.

Blomkamp E. Systemic design practice for participatory policymaking. Policy Design and Practice. 2022 Jan 2;5(1):12–31.

Brennan SE, Cumpston M, Misso ML, McDonald S, Murphy MJ, Green SE. Design and formative evaluation of the Policy Liaison Initiative: a long-term knowledge translation strategy to encourage and support the use of Cochrane systematic reviews for informing health policy. Evidence & Policy. 2016 Jan;12(1):25–52.

Campos PA, Reich MR. Political Analysis for Health Policy Implementation. Health Systems & Reform. 2019 Jul 3;5(3):224–35.

Chahine T. Toward an Understanding of Public Health Entrepreneurship and Intrapreneurship. Front Public Health. 2021 Apr 9;9:593553.

Chao K, Sarker MNI, Ali I, Firdaus RBR, Azman A, Shaed MM. Big data-driven public health policy making: Potential for the healthcare industry. Heliyon. 2023 Sep;9(9):e19681.

Ferrinho P, Lehman U, Kovacs E, Poz MD. Relevant HRH leadership during public health emergencies. Hum Resour Health. 2022 Dec;20(1):28.

Gershuni O, Czabanowska K, Burazeri G, Bjegovic-Mikanovic V, Juszczyk G, Myrup AC, et al. Aligning Best Practices: A Guiding Framework as a Valuable Tool for Public Health Workforce Development with the Example of Ukraine. IJERPH. 2021 Sep 1;18(17):9246.

Hallsworth M. Policy-Making in the Real World. Political Insight. 2011 Apr;2(1):10–2.

Hassanzadeh A, Namdarian L, Majidpour M, Elahi S. Developing a model to evaluate the impacts of science, technology and innovation foresight on policy-making. Technology Analysis & Strategic Management. 2015 Apr 21;27(4):437–60.

Hoppe R. Rules-of-thumb for problem-structuring policy design. Policy Design and Practice. 2018 Jan 2;1(1):12–29.

Houngbo PT, De Cock Buning T, Bunders J, Coleman HLS, Medenou D, Dakpanon L, et al. Ineffective Healthcare Technology Management in Benin’s Public Health Sector: The Perceptions of Key Actors and Their Ability to Address the Main Problems. Int J Health Policy Manag. 2017 Feb 20;6(10):587–600.

Jewell CJ, Bero LA. “Developing Good Taste in Evidence”: Facilitators of and Hindrances to Evidence‐Informed Health Policymaking in State Government. Milbank Quarterly. 2008 Jun;86(2):177–208.

Koduah A, Agyepong IA, Van Dijk H. ‘The one with the purse makes policy’: Power, problem definition, framing and maternal health policies and programmes evolution in national level institutionalised policy making processes in Ghana. Social Science & Medicine. 2016 Oct;167:79–87.

Könnölä T, Scapolo F, Desruelle P, Mu R. Foresight tackling societal challenges: Impacts and implications on policy-making. Futures. 2011 Apr;43(3):252–64.

Koon AD, Windmeyer L, Bigdeli M, Charles J, El Jardali F, Uneke J, et al. A scoping review of the uses and institutionalisation of knowledge for health policy in low- and middle-income countries. Health Res Policy Sys. 2020 Dec;18(1):7.

Lee W, Choi S. Educational strategies to encourage participation in health policy for nurses: A systematic review. Nurse Education Today. 2022 Apr;111:105310.

Lesch M, McCambridge J. Coordination, framing and innovation: the political sophistication of public health advocates in Ireland. Addiction. 2021 Nov;116(11):3252–60.

Loncarevic N, Andersen PT, Leppin A, Bertram M. Policymakers’ Research Capacities, Engagement, and Use of Research in Public Health Policymaking. IJERPH. 2021 Oct 20;18(21):11014.

Mahdavi M, Sajjadi Khasraghi J, Sajadi HS, Yazdizadeh B, Nikooee S, Ehsani-Chimeh E, et al. Developing Framework and Strategies for Capacity Building to Apply Evidence-Informed Health Policy-Making in Iran: Mixed Methods Study of SAHSHA Project. Int J Health Policy Manag. 2021 Oct 11;1.

Mählmann L, Reumann M, Evangelatos N, Brand A. Big Data for Public Health Policy-Making: Policy Empowerment. Public Health Genomics. 2017;20(6):312–20.

Mayer IS, Van Daalen CE, Bots PWG. Perspectives on Policy Analysis: A Framework for

Understanding and Design. In: Thissen WAH, Walker WE, editors. Public Policy Analysis

[Internet]. Boston, MA: Springer US; 2013 [cited 2025 Mar 21]. p. 41–64. (International

Series in Operations Research & Management Science; vol. 179).

Oronje RN, Murunga VI, Zulu EM. Strengthening capacity to use research evidence in health sector policy-making: experience from Kenya and Malawi. Health Res Policy Sys. 2019 Dec;17(1):101.

Pirani S, De Pinho H, Arana M, Bhaskar S, Hale B, Murrman M. Incorporating Systems Thinking Approaches Into Practice-Based Training to Strengthen Policy-Making Skills. Journal of Public Health Management and Practice. 2022 Sep;28(Supplement 5):S249–53.

Putturaj M, Bhojani U, Rao N, Marchal B. Decoding the black box of health policy implementation: A case of regulating private healthcare establishments in southern India. Natl Med J India. 2021;34(2):100.

Ramezani M, Takian A, Bakhtiari A, Rabiee HR, Ghazanfari S, Mostafavi H. The application of artificial intelligence in health policy: a scoping review. BMC Health Serv Res. 2023 Dec 15;23(1):1416.

Sanderson I. Intelligent Policy Making for a Complex World: Pragmatism, Evidence and Learning. Political Studies. 2009 Dec;57(4):699–719.

Schultz S, Zorbas C, Peeters A, Yoong S, Backholer K. Strengthening local government policies to address health inequities: perspectives from Australian local government stakeholders. Int J Equity Health. 2023 Jun 21;22(1):119.

Shearer JC. Policy entrepreneurs and structural influence in integrated community case management policymaking in Burkina Faso. Health Policy Plan. 2015 Dec;30(suppl 2):ii46–53.

Shroff Z, Aulakh B, Gilson L, Agyepong IA, El-Jardali F, Ghaffar A. Incorporating research evidence into decision-making processes: researcher and decision-maker perceptions from five low- and middle-income countries. Health Res Policy Sys. 2015 Dec;13(1):70.

Sohn J. Navigating the politics of evidence-informed policymaking: strategies of influential policy actors in Ontario. Palgrave Commun. 2018 Apr 24;4(1):49.

Stoto MA, Nelson C, Savoia E, Ljungqvist I, Ciotti M. A Public Health Preparedness Logic Model: Assessing Preparedness for Cross-border Threats in the European Region. Health Security. 2017 Oct;15(5):473–82.

Trott A, Srinivasa S, van der Wal D, Haneuse S, Zheng S. Building a Foundation for Data-Driven, Interpretable, and Robust Policy Design using the AI Economist [Internet]. arXiv; 2021 [cited 2025 Mar 21]. Available from: <https://arxiv.org/abs/2108.02904>

Uneke CJ, Sombie I, Uro-Chukwu HC, Mohammed YG, Johnson E. Promoting evidence informed policymaking for maternal and child health in Nigeria: lessons from a knowledge translation workshop. Health Promot Perspect. 2017 Nov 6;8(1):63–70.

Waqa G, Mavoa H, Snowdon W, Moodie M, Nadakuitavuki R, Mc Cabe M, et al. Participants’ perceptions of a knowledge-brokering strategy to facilitate evidence-informed policy-making in Fiji. BMC Public Health. 2013 Aug 7;13(1):725.

Wardle C, Derakhshan H. Information disorder: Toward an interdisciplinary framework for research and policy making [Internet]. Strasbourg: Council of Europe; 2017 p. 109. (Council of Europe Report). Report No.: DGI(2017)09. Available from: <https://edoc.coe.int/en/media/7495-information-disorder-toward-an-interdisciplinary-framework-for-research-and-policy-making.html>

Wheat ID. What can system dynamics learn from the public policy implementation literature? Syst Res. 2010 Jul;27(4):425–42.

Wu X, Ramesh M, Howlett M. Policy capacity: A conceptual framework for understanding policy competences and capabilities. Policy and Society. 2015 Sep 1;34(3–4):165–71.

Wu X, Ramesh M, Howlett M. Policy Capacity: Conceptual Framework and Essential Components. In: Wu X, Howlett M, Ramesh M, editors. Policy Capacity and Governance [Internet]. Cham: Springer International Publishing; 2018 [cited 2025 Mar 21]. p. 1–25. Available from: <http://link.springer.com/10.1007/978-3-319-54675-9_1>
