# Supplementary material for: Defining Competencies for Policymaking in Public Health: A Scoping Review on the State-of- The-Art
Source: Public Health Rev. 2026 May 14;47:1609031. doi: 10.3389/phrs.2026.1609031 (PMC13217404; doi:10.3389/phrs.2026.1609031)
Supplement: Supplementary file 3 [file Table3.docx]

**Appendix 3. Data extraction template**

| No. |  |
| --- | --- |
| **Reviewer’s Initials** |  |
| **Article Reference (APA reference style)** |  |
| **Year of Publication** |  |
| **Abstract** |  |
| **Article Type:**  E.g.  - Systematic Review  - Scoping Review  - Extra org. study  - WHO study |  |
| **Country case(s):** |  |
| **Level(s) of policymaking addressed:**  E.g.  - International (IOs)  - Supranational (e.g. EU)  - National  - Sub-national (Regions, Municipalities etc.) |  |
| **Target Group:**  Competencies refer to:  - Policymakers (as individuals) in public health AND/OR  - Policymaking institutions (as collectives) |  |
| **Specific policymaking stage(s) addressed:**  E.g.  - Agenda setting  - Policy formulation/design  - Policy selection  - Policy implementation  - Policy evaluation |  |
| **Core competencies addressed/ identified (by policymaking stage, if differentiated)***^[[1]](#footnote-1)^ |  |

Source: Own presentation.

1. Note that in many cases competencies may not be explicitly stated in the article but can be derived from the facilitators and/or barriers to their use identified in the next two cells of the template. [↑](#footnote-ref-1)
